# Supplementary material for: Generation of a Pure Culture of Neuron-like Cells with a Glutamatergic Phenotype from Mouse Astrocytes
Source: Biomedicines. 2022 Apr 18;10(4):928. doi: 10.3390/biomedicines10040928 (PMC9031297; doi:10.3390/biomedicines10040928)
Supplement: Supplementary file 1 [file biomedicines-10-00928-s001.zip › biomedicines-1666034-supplementary.pdf]

Supplementary Information

Generation of a pure culture of neuron-like cells with a glutamatergic phenotype from mouse astrocytes

Gary Stanley Fernandes[1], Rishabh Singh Deo[1], and Kyeong Kyu Kim[1, *]

[1] Department of Precision Medicine, Graduate School of Basic Medical Science (GSBMS), Institute for Anti-microbial Resistance Research and Therapeutics, Sungkyunkwan University School of Medicine, Suwon, 16419, Korea

* Correspondence: kyeongkyu@skku.edu (K.K.K.); Tel.: +82-31-299-6152

*To whom all correspondence should be addressed:

Tel: 82-31-299-6136

Fax: 82-31-299-6159

E-mail: kyeongkyu@skku.edu

Contents:

Supplementary Figures

Figure S1.

Figure S2

Figure S3

Figure S4

Figure S5

Supplementary Tables

Table S1: List of primers used in RT-qPCR analysis.

Table S2: List of antibodies used in this study.

Table S3: List of conversions of ectodermal lineage cells into neurons using chemical cocktail.

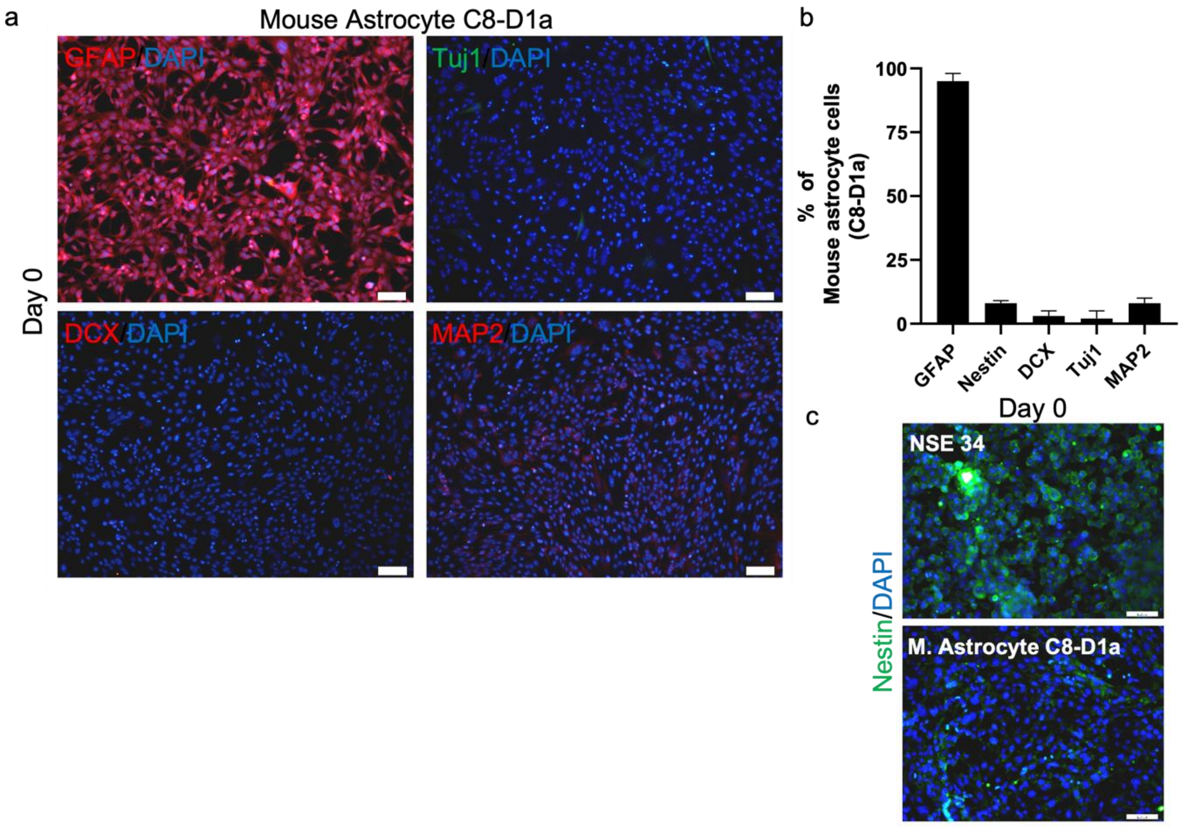

Figure S1. a) Immunofluorescence labeling of mouse astrocytes C8-D1a. Scale bar, 50 μm. b) Percentage of cells positive for the astrocytic marker GFAP, the stem cell marker nestin, and the neuronal markers DCX, Tuj1, and MAP2. c) Immunofluorescence labeling with the stem cell marker nestin and NSC-34 as a positive control on mouse astrocytes C8-D1a. Scale bar, 20 μm.

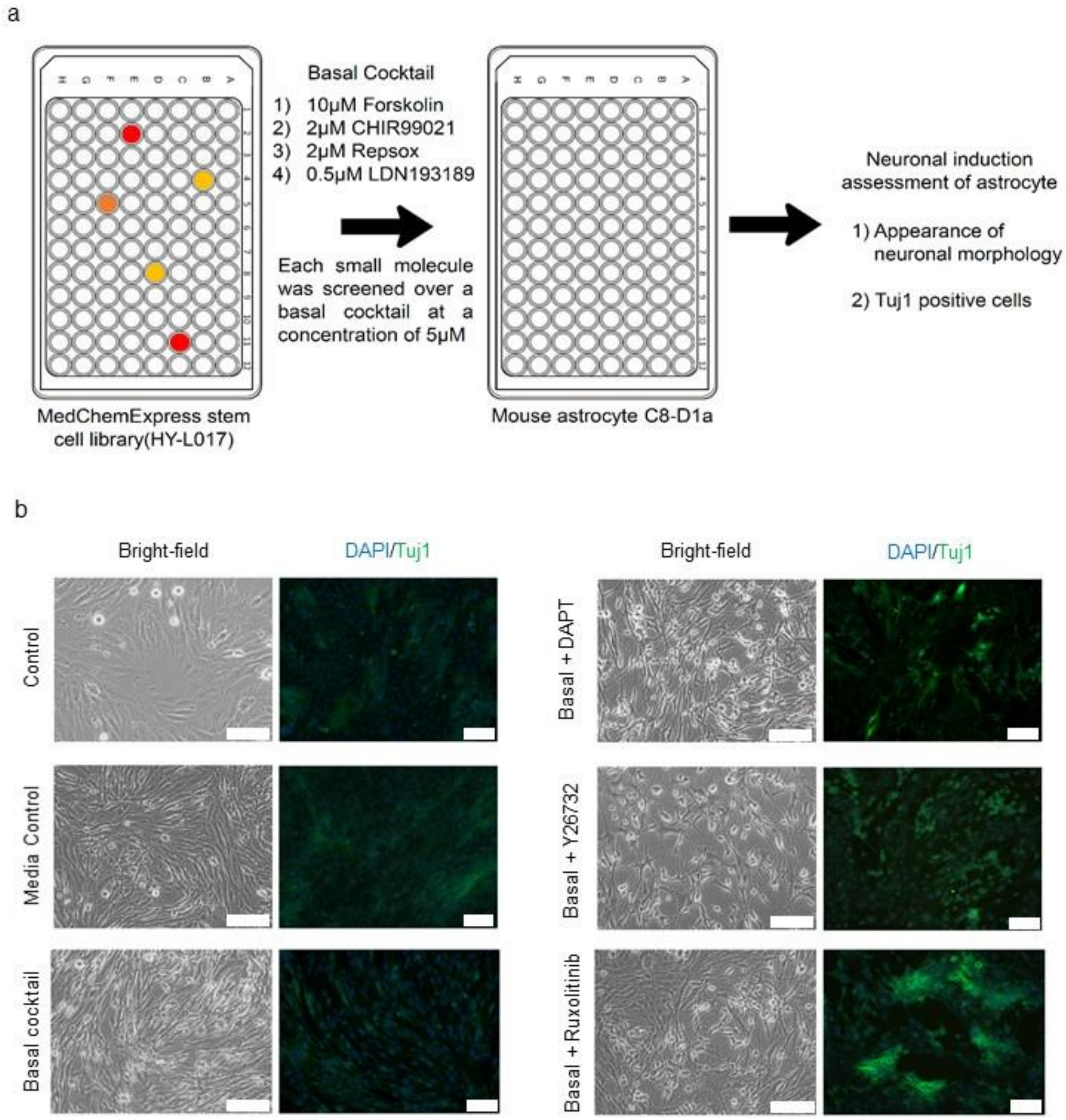

Figure S2. a) Schematic design for screening small molecules from the MedChemExpress library (HY-L017). b) Comparison of bright-field images and Tuj1$^+$ immunostaining images from positive hits in the presence of the basal cocktail, medium control, and control. Scale bar, 100 μm for bright-field, 50 μm for immunostaining images.

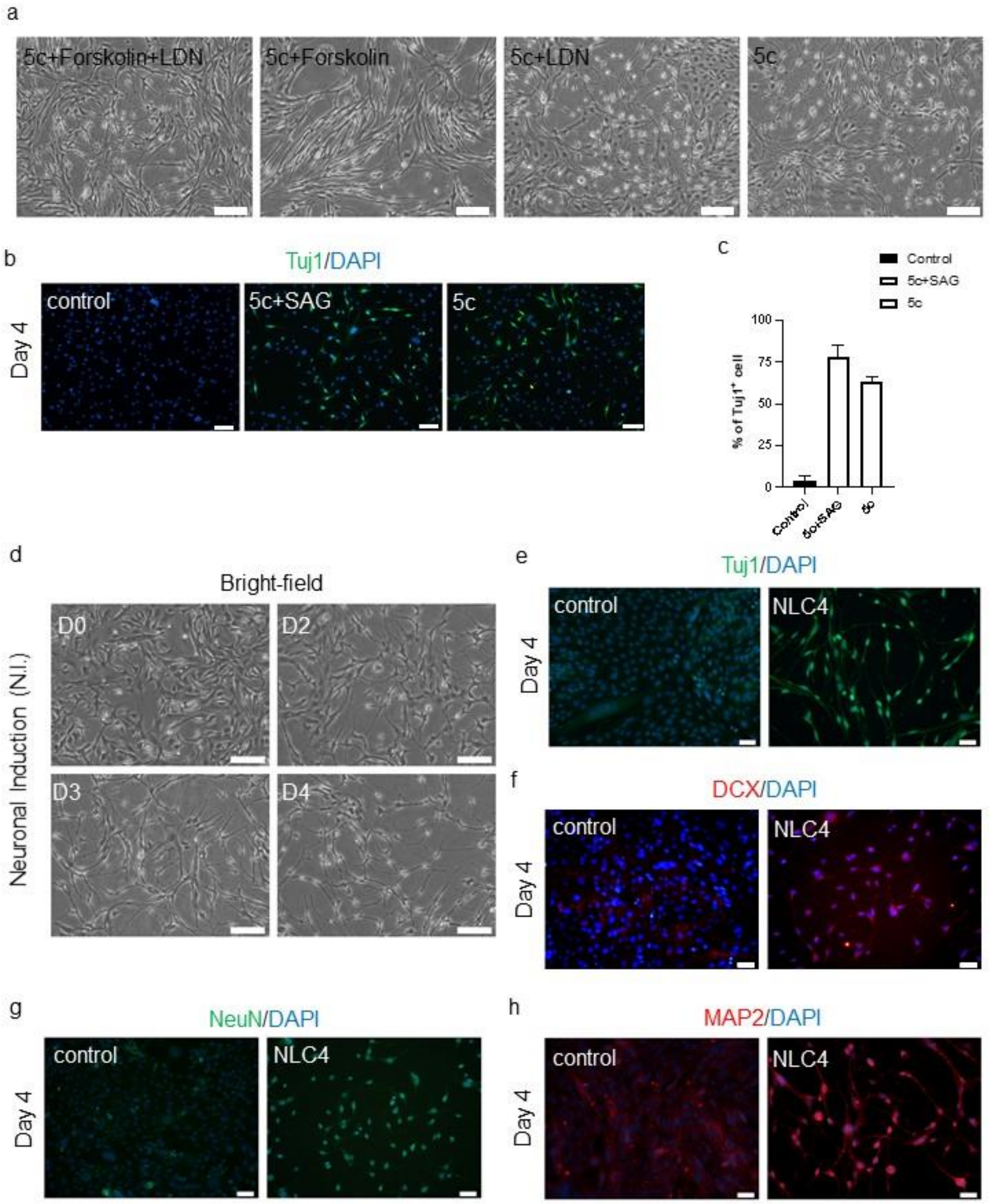

Figure S3. a) Immunostaining of four-day-old neuronally induced astrocytes to investigate the addition of SAG to the 5C cocktail. Scale bar, 100 µm. b) Tuj1^{+} cell count of the 5C and 5C+SAG cocktails. c) Quantification of Tuj1^{+} cells in the presence and absence of SAG. d) Representative bright-field image of mouse

astrocytes C8-D1a during four days of exposure to our neuronal induction cocktail. Scale bar, 100 μm. e) Immunostaining with the neuronal marker Tuj1 in four-day-old neuronally induced mouse astrocytes C8-D1a. Scale bar, 50 μm. f) Immunostaining with the neuronal marker DCX in four-day-old neuronally induced mouse astrocytes C8-D1a. Scale bar, 50 μm. g) Immunostaining with the neuronal marker NeuN in four-day-old neuronally induced mouse astrocytes C8-D1a. Scale bar, 50 μm. h) Immunostaining with the neuronal marker MAP2 in four-day-old neuronally induced mouse astrocytes C8-D1a cell-line. Scale bar, 50 μm.

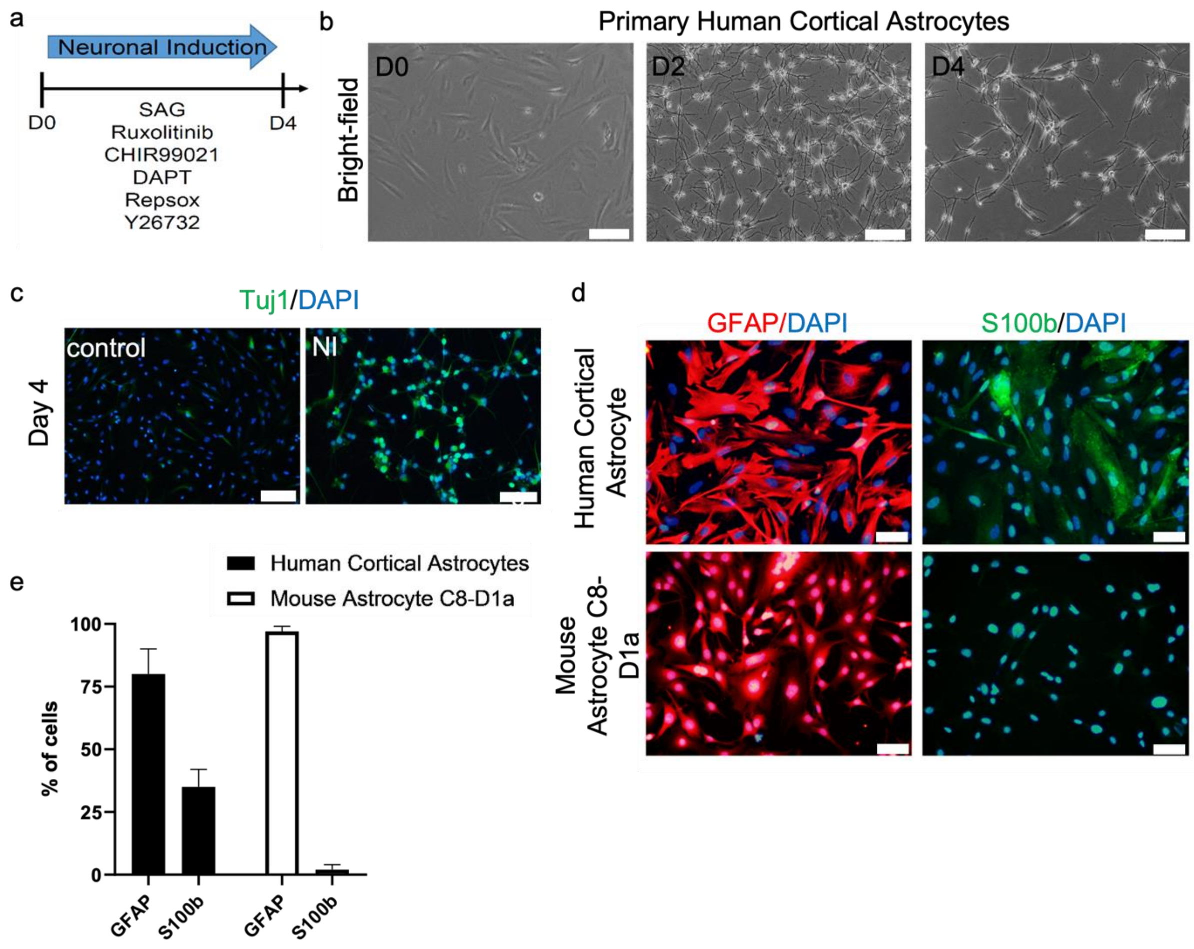

Figure S4. a) Schematic design of the protocol for converting human cortical astrocytes into neurons. b) Representative bright-field image of human cortical astrocytes during four days of exposure to our neuronal induction cocktail. Scale bar, 100 µm. c) Immunostaining of neuronal induced (NI) primary human cortical astrocytes with neuronal marker Tuj1 upon treated with NIM for four days. Scale bar, 50 µm. d) Immunofluorescence labeling with GFAP and S100b in mouse astrocytes C8-D1a and human cortical astrocytes. Scale bar, 50 µm. e) Percentage of cells positive for the astrocytic markers GFAP and S100b.

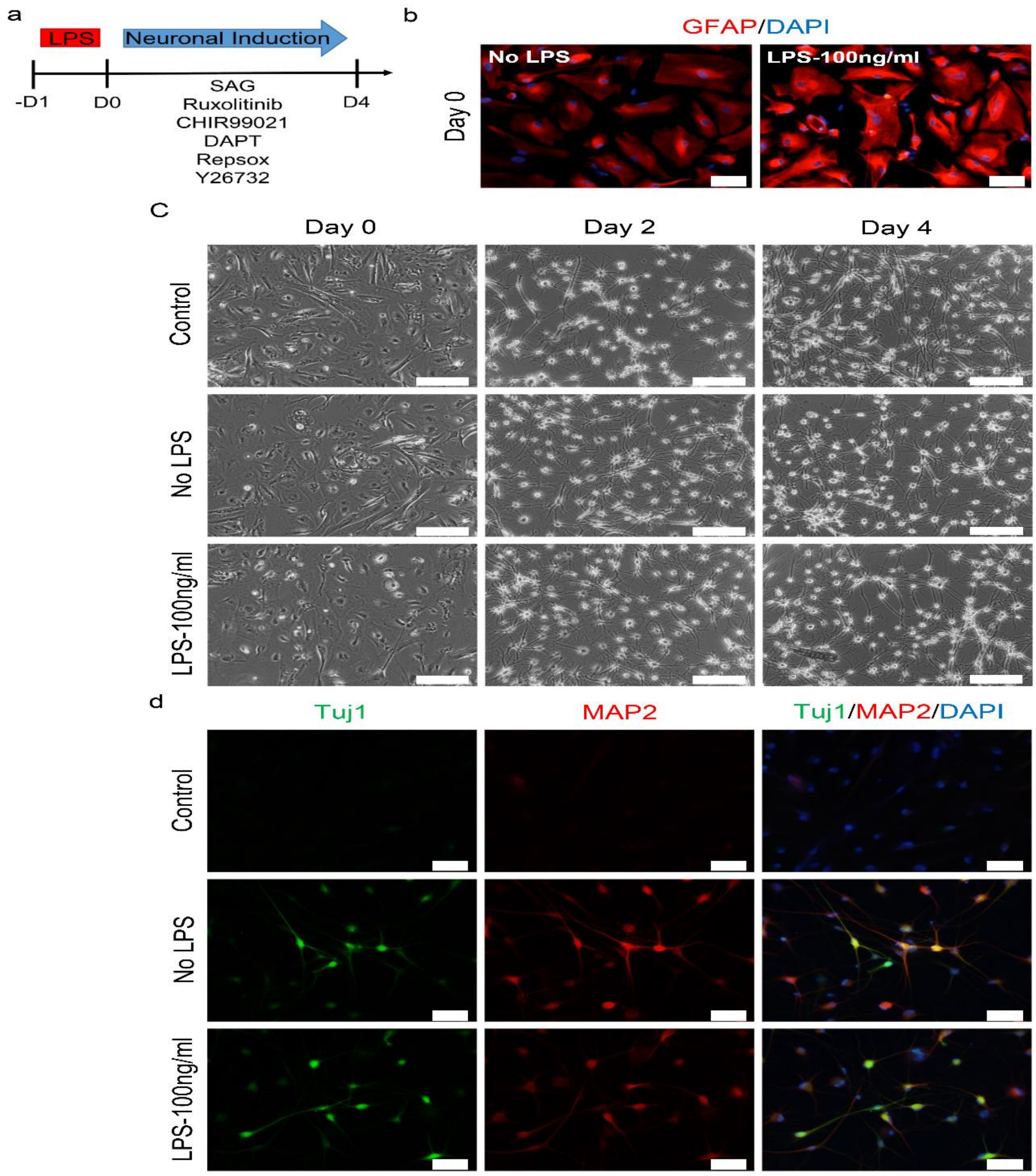

Figure S5 a) Schematic design for assessing neuronal reprogramming potential of our chemical cocktail on LPS-induced human cortical reactive astrocytes. b) Immunostaining of chemically induced astrocytes with astrocyte marker GFAP pretreated with or without LPS. Scale bar, 50 μm. c) Representative bright-field images of chemically induced astrocytes over four days of exposure to our neuronal induction cocktail pretreated with or without LPS. Scale bar, 100 μm. d) Immunofluorescence labeling chemically induced astrocytes with Tuj1and MAP2 after four days of exposure in neuronal induction cocktail pretreated with or without LPS. Scale bar, 50 μm.

Supplementary Tables

Table S1: List of primers used in RT-qPCR analysis.

Gene Name	Position	Sequence
NeuroD1	5’ Prime	ACGCAGAAGGCAAGGTGTCC
	3’ Prime	GTTCCTCGTCCTGAGAACTG
Ngn2	5’ Prime	TGGCTGGCATCTGCTCTATT
	3’ Prime	TAGGCATTGTGACGAATCTG
Ascl1	5’ Prime	ACTTGAACTCTATGGCGGGTT
	3’ Prime	CCAGTTGGTAAAGTCCAGCAG
Tbr1	5’ Prime	ACAATGGGCAGATGGTGGTT
	3’ Prime	GTGTCCTCTGTGCCATCCTC
Lmx1a	5’ Prime	GCAAAGGGGACTATGAGAAGGA
	3’ Prime	CGTTTGGGGCGCTTATGGT
Nurr1	5’ Prime	ACCACTCTTCGGGAGAATACA
	3’ Prime	GGCATTTGGTACAAGCAAGGT
DCX	5’ Prime	TTCAGGACCACAAGCAATGA
	3’ Prime	GGAAACCGGAGTTGTCAAAA
NeuN	5’ Prime	ACAGACAACCAGCAACTCCA
	3’ Prime	CCGAATTGCCCGAACAT
MAP2a	5’Prime	AACCAATTCGCAGAGCAGGA
	3’Primer	GGGAGTTCCAGGGGTGATTG
Syn1	5’Prime	TCTTCCTCCAACCTCCACTCCT

	3'Primer	TGGGCCTTTGCTTGTTTATTTT
vGLUT1	5'Prime	TGTTGGGTTTGGGGATT
	3'Primer	AGGTTTTATGCTTTGCACTTA
GAD67	5'Prime	CACAAACTCAGCGGCATAGA
	3'Primer	CTGGAAGAGGTAGCCTGCAC
Th	5'Prime	CACTATGCCCACCCCCAG
	3'Primer	CGCCGTCCAATGAACCTT
Achat	5' Primer	ACTGGGTGTCTGAGTACTGG
	3' Primer	TTGGAAGCCATTTTGACTAT
Gapdh	5' Primer	AGGTCGGTGTGAACGGATTTG
	3' Primer	TGTAGACCATGTAGTTGAGGTCA

Table S2: List of antibodies used in this study.

Primary antibody	Company (Catalog No.)	Dilution ratio	Species	Secondary antibody	Company (catalog No.	Dilution ratio
Anti-Nestin	Genetex (GTX630201)	1:300	Mouse	Goat Anti-Mouse IgG H&L(Alexa Fluor 488)	Thermofisher (A-11029)	1:500
Anti-GFAP	Novos Biological (NB300-141)	1:300	Rabbit	Goat Anti-Mouse IgG H&L(Alexa Fluor 546)	Thermofisher (A-11010)	1:500
Anti-Tuj1	R&D SYSTEMS (MAB1195)	1:300	Mouse	Goat Anti-Mouse IgG H&L(Alexa Fluor 488)	Thermofisher (A-11029)	1:500
Anti-Tuj1	BioLegend (802001)	1:300	Rabbit	Goat Anti-Mouse IgG H&L(Alexa Fluor 546)	Thermofisher (A-11010)	1:500
Anti-NeuN	Millipore (MAB377)	1:100	Mouse	Goat Anti-Mouse IgG H&L(Alexa Fluor 488)	Thermofisher (A-11029)	1:500
Anit-DCX	Abcam (ab18723)	1:300	Rabbit	Goat Anti-Mouse IgG H&L(Alexa Fluor 546)	Thermofisher (A-11010)	1:500
Anti-MAP2	Millipore (ab5622)	1:300	Rabbit	Goat Anti-Mouse IgG H&L(Alexa Fluor 546)	Thermofisher (A-11010)	1:500
Anit-Syn1	Millipore (ab1543)	1:300	Rabbit	Goat Anti-Mouse IgG H&L(Alexa Fluor 546)	Thermofisher (A-11010)	1:500
Anti-vGLUT1	Millipore (MAB5502)	1:300	Mouse	Goat Anti-Mouse IgG H&L(Alexa Fluor 488)	Thermofisher (A-11029)	1:500
Anti-GAD67	Millipore (MAB5406)	1:300	Mouse	Goat Anti-Mouse IgG H&L(Alexa Fluor 488)	Thermofisher (A-11029)	1:500
Anti-TH	Millipore	1:300	Mouse	Goat Anti-Mouse IgG	Thermofisher (A-11029)	1:500

	(MAB318)			H&L(Alexa Fluor 488)		
Anti-S100b	Thermofisher (MA5-12969)	1:300	Mouse	Goat Anti-Mouse IgG H&L(Alexa Fluor 488)	Thermofisher (A-11029)	1:500

Table S3: List of conversions of ectodermal lineage cells into neurons using chemical cocktail.

Starting cell type	Generate cells	Chemical cocktail used	Reference
Müller cells	Neuron-like cells	Forskolin, ISX9, CHIR99021, I-BET151, and Y-27632	[68]
Apical papilla stem cells	Neuron-like cells	VPA, CHIR99021, RepSox, Forskolin, SP600125, GO6983, Y-27632	[69]
